# O.5.3-7 Evaluation of implementation strategies promoting Interprofessional Collaboration Practices among social and health professionals involved in fall prevention in the community

**DOI:** 10.1093/eurpub/ckad133.258

**Published:** 2023-09-11

**Authors:** Saskia te Velde, Meike van Scherpenseel, Lidia van Veenendaal, Sabine de Vries, Rixt Zuidema

**Affiliations:** HU University of Applied Sciences Utrecht, The Netherlands; saskia.tevelde@hu.nl; HU University of Applied Sciences Utrecht, The Netherlands; saskia.tevelde@hu.nl; HU University of Applied Sciences Utrecht, The Netherlands; saskia.tevelde@hu.nl; HU University of Applied Sciences Utrecht, The Netherlands; saskia.tevelde@hu.nl; HU University of Applied Sciences Utrecht, The Netherlands; saskia.tevelde@hu.nl

## Abstract

**Purpose:**

Fall prevention practices (FPPs), including exercised-based programs, are effective in reducing fall rates among community-dwelling older adults, but they are not implemented enough to reach their full potential. Given the multifactorial nature of falls, these FPPs should have an integrated approach where multiple social and health professionals achieve good Interprofessional Collaboration Practices (ICP). The aim of the current study is to evaluate implementation strategies promoting ICP among professionals involved in fall prevention.

**Methods:**

We performed longitudinal process evaluations with a mixed-methods approach including document study, dynamic learning agenda (DLA), and a social network analysis (SNA) survey in four communities in the region of Utrecht, the Netherlands. Professionals involved in fall prevention participated in regular meetings and responded to SNA surveys. This survey was distributed at baseline, after six, 12 and 18 months, which measured, among other things, the number of links between professionals. Evaluation whether the implementation strategies, such as training, providing a social care map and establishing referral pathways, improved ICP were guided by The Conceptual Framework ICP.

**Results:**

Data collection is ongoing and results will be updated. Until now, the SNA shows that in all communities physical therapists and primary care and community nurses play a central role, and that over time more professionals were included in the network. The DLA showed that changes in barriers for ICP differed per community; in one community time and budget remained main barriers, while in another community barriers were successfully transformed in facilitators. For example, initially professionals did not know each other well, but after using the implementation strategies they understood each other's role better, which highly facilitates efficient ICP.

**Conclusions:**

The effect of implementation strategies are dependent on local contexts. Furthermore, continuous evaluations, such as insight in present barriers and facilitators, and reflections are necessary to select the implementation strategies that fit best to the current context. These findings will help professionals involved in fall prevention to implement and upscale FPPs in the community.

**Support/Funding Source:**

This research is co-financed by Regieorgaan SIA, part of the Netherlands Organisation for Scientific Research (NWO) (project number RAAK.PRO03.099).

